# Omega-3 Fatty Acid Supplementation Appears to Attenuate Particulate Air Pollution–Induced Cardiac Effects and Lipid Changes in Healthy Middle-Aged Adults

**DOI:** 10.1289/ehp.1104472

**Published:** 2012-04-19

**Authors:** Haiyan Tong, Ana G. Rappold, David Diaz-Sanchez, Susan E. Steck, Jon Berntsen, Wayne E. Cascio, Robert B. Devlin, James M. Samet

**Affiliations:** 1 Environmental Public Health Division, National Health and Environmental Effects Research Laboratory, U.S. Environmental Protection Agency, Research Triangle Park, North Carolina, USA; 2 Department of Epidemiology and Biostatistics, University of South Carolina, Columbia, South Carolina, USA; 3 TRC Environmental Corporation, Raleigh, North Carolina, USA

**Keywords:** cardiac effect, controlled exposure, lipid change, middle-aged healthy human volunteers, omega-3 fatty acid, particulate matter air pollution

## Abstract

Background: Air pollution exposure has been associated with adverse cardiovascular health effects. Findings of a recent epidemiological study suggested that omega-3 fatty acid (fish oil) supplementation blunted cardiac responses to air pollution exposure.

Objectives: We conducted a randomized, controlled exposure study to evaluate the efficacy of fish oil supplements in attenuating adverse cardiac effects of exposure to concentrated ambient fine and ultrafine particulate matter (CAP).

Methods: Twenty-nine healthy middle-aged participants (mean, 58 ± 1 years of age) were supplemented in a randomized, double-blinded manner with 3 g/day of either fish oil or olive oil for 4 weeks before sequential chamber exposure to filtered air and CAP (mean mass concentration 278 ± 19 µg/m^3^) for 2 hr. Cardiac responses were assessed by comparing time and frequency domain changes in heart rate variability (HRV) and electrocardiographic repolarization changes measured before, immediately after, and 20 hr after exposure. Changes in plasma lipids were also evaluated at these time points.

Results: Fish oil supplementation appeared to attenuate CAP-induced reductions in high-frequency/low-frequency ratio, as well as elevations in normalized low-frequency HRV and prolongation of the QT interval corrected for heart rate (QTc). Very low-density lipoprotein and triglyceride concentrations increased significantly immediately after exposure to CAP in participants supplemented with olive oil, but not in those supplemented with fish oil.

Conclusions: Exposure of healthy middle-aged adults to CAP for 2 hr induced acute cardiac and lipid changes after supplementation with olive oil, but not fish oil. Our findings suggest that omega-3 fatty acid supplements offer protection against the adverse cardiac and lipid effects associated with air pollution exposure.

Ambient air pollution exposure is a major environmental risk to human health. The World Health Organization (WHO) has estimated that air pollution causes approximately two million deaths per year worldwide (WHO 2011), and the number is climbing. Epidemiological and experimental studies have demonstrated positive associations between adverse cardiopulmonary effects and acute and chronic exposure to levels of ambient air pollution currently found in major metropolitan areas [Brook et al. 2010; U.S. Environmental Protection Agency (EPA) 2009]. Short-term elevations in ambient particulate matter (PM) concentrations are associated with acute coronary syndrome, stroke, venous thrombosis, arrhythmia, and worsening of heart failure, particularly in certain at-risk populations (U.S. EPA 2009). Cohort studies have reported associations between PM exposure and modulation of the cardiac autonomic nervous system input leading to alterations in heart rate variability (HRV) (Gold et al. 2000; He et al. 2011; Pope et al. 2004). Our previous controlled human exposure studies have shown that concentrated ambient PM exposure changed HRV in healthy adults (Devlin et al. 2003; Samet et al. 2009). Previous studies have also linked PM exposure with elevated blood lipid levels in both humans and animals (Sun et al. 2005; Yeatts et al. 2007).

Ambient PM is a complex mixture of particles of varying size that are classified as coarse (PM_2.5–10_; 10 µm > PM > 2.5 µm in aerodynamic diameter), fine (PM_2.5;_ PM < 2.5 µm), and ultrafine (PM_0.1_; PM < 0.1 µm) particles. The strongest associations between adverse health effects and particulate air pollution are observed with PM_2.5_ (Schwartz et al. 1996). However, PM_0.1_ is not routinely measured at state monitoring sites nor is it homogenously dispersed throughout an airshed, making it difficult to ascertain the toxicity of these particles in epidemiological studies. However, clinical and toxicological studies have reported that PM_0.1_ can pass through the lung epithelial cell barrier and enter the circulatory system, where it may directly affect the function of cells or organs (Brook et al. 2010; Nemmar et al. 2004). Therefore, in this study, participants were exposed to a combination of concentrated PM_2.5_ and PM_0.1_ ambient air particles (CAP).

Although air pollution regulations have resulted in dramatic decreases in PM levels over the past few decades, tens of millions of persons in the United States are still exposed to levels of PM that are higher than the current U.S. EPA standards (U.S. EPA 2009). If dietary supplements or pharmacological agents can blunt the adverse health effects of air pollution exposure, populations residing in highly polluted areas might be afforded some protection. A number of studies conducted over the last three decades have shown that the omega-3 polyunsaturated fatty acids (n-3 FA), eicosapentaenoic acid and docosahexaenoic acid (DHA), can reduce the risk of cardiovascular events after myocardial infarction (O’Keefe et al. 2006; Saravanan et al. 2010) as well as reduce atrial fibrillation recurrences in patients with persistent atrial fibrillation (Nodari et al. 2011). In general, n-3 FA have several potentially cardioprotective benefits, including suppression of arrhythmias and modulation of autonomic function in addition to antithrombotic, anti-inflammatory, and vasodilatory effects (Lee et al. 2009). Chronically decreased HRV has been used to predict an increased risk of cardiovascular morbidity and mortality (Kleiger et al. 1987; Lahiri et al. 2008; Tsuji et al. 1996), and several studies suggest that the intake of n-3 FA may improve autonomic function, including an increase in vagal activity and baroreflex sensitivity (Mozaffarian et al. 2008; O’Keefe et al. 2006).

A recent cohort panel study reported that the high- and low-frequency (HF and LF) components of HRV were increased among elderly participants after n-3 FA supplementation (Holguin et al. 2005) and that HRV decline associated with PM_2.5_ exposure before supplementation was not observed after n-3 FA supplementation (Romieu et al. 2005). To our knowledge, no controlled human exposure studies have been conducted to investigate the protective effect of n-3 FA supplementation against the adverse cardiovascular effects of air pollutant exposure. We designed this study to determine whether the cardiovascular effects caused by exposure to CAP could be blunted by supplementation with n-3 FA, compared with supplementation with oil devoid of n-3 FA (olive oil). We report here that supplementation of n-3 FA attenuated some adverse effects on autonomic function, ventricular repolarization, and lipid changes caused by exposure to CAP in healthy middle-aged adults.

## Methods

*Study participants.* Twenty-nine participants ranging from 50 to 72 years of age (mean 58 ± 1 years) were enrolled in the study. They were nonsmokers for at least 1 year, with no history of heart disease, uncontrolled hypertension [mean blood pressure (BP): 123 ± 3/77 ± 2 mmHg], pulmonary disease, diabetes mellitus, hypercholesterolemia, or active allergy. Participants were not taking β-adrenergic receptor blockers, n-3 FA, anti-inflammatory drugs, or antioxidant supplements (such as beta-carotene, selenium, vitamin C, or vitamin E). All participants were instructed to refrain from using any pain medications for 2 weeks before each exposure. They were also asked to abstain from alcohol and caffeine and to adhere to a low-fat diet for 24 hr before exposures. The Biomedical Institutional Review Board at the University of North Carolina–Chapel Hill and the U.S. EPA approved the study protocol, recruitment materials, and consent forms. All study participants gave informed consent and received monetary compensation for their participation.

**Figure 1:**
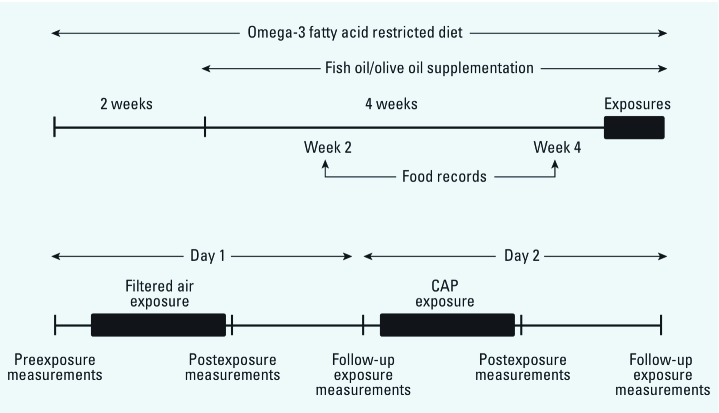
Schematic representation of the study design.

*Study design.* This study was conducted from July 2009 to August 2010. All exposures were conducted at the U.S. EPA Human Studies Facility on the University of North Carolina–Chapel Hill campus. A diagram of the study design is shown in Figure 1. Sixteen participants were assigned to receive 3 g/day (three 1-g capsules daily) of marine-derived n-3 FA (fish oil; FO), and 13 participants received 3 g/day (three 1-g capsules daily) of olive oil (OO) for 28 days before the filtered air exposure day. FO and OO assignments were made using a randomized, double-blinded study design. Each participant was exposed first to filtered air and then to CAP on the next day. The exposures were conducted at the same time of the day and same day of the week. Participants were exposed for 2 hr through a face mask in an exposure chamber in which temperature and humidity were controlled. Participants remained at rest in a seated position throughout the exposure.

The following tests were done on each participant beginning at approximately 0800 hours (2 hr before exposure to filtered air): Venous blood was collected (120 min before exposure); Holter electrodes were applied and HRV and repolarization data obtained (105 min before exposure); and brachial artery diameter was measured by ultrasound (60 min before exposure). The ultrasound measurements will be reported elsewhere. The same tests were done immediately after the 2-hr air exposure (Post), and again the next morning at approximately 0800 hours (Follow-up). These latter measurements also served as the preexposure values for the CAP exposure. At approximately 1000 hours on the second day, participants were exposed to CAP for 2 hr. Post and Follow-up measurements were obtained immediately after exposure and again beginning at 0800 hours the next morning. The participants wore a portable ambulatory Holter device for the entire 48-hr period and time domain HRV variables were calculated from the two 24-hr periods.

*Dietary supplementations.* All participants were asked to refrain from food containing n-3 FA for 2 weeks before and 4 weeks during the dietary supplementation period. Participants were also asked to keep 3-day food records during the 2nd and 4th weeks of the supplementation period to assess compliance with the dietary restrictions. Nutrition Data System for Research software (version 2011; Nutrition Coordinating Center, University of Minnesota, Minneapolis, MN) was used to analyze the food records and estimate intakes of nutrients that may confound n-3 FA measurements. Each 1,000-mg FO capsule contained approximately 65% n-3 FA (410 mg eicosapentaenoic acid and 274 mg DHA). Each 1,000-mg OO capsule contained < 1% n-3 FA (73% oleic acid and 12% palmitic acid). Pharmavite, LLC (Mission Hills, CA) provided the FO and OO supplements. The ratios of the major plasma fatty acids were measured at the end of the supplementation period to determine whether ratios were consistent with expectations for the FO and OO groups.

*Controlled exposure.* CAP was generated as described previously (Samet et al. 2009) by drawing ambient air from above the roof of the Human Studies Facility and passing the air through a 2-stage aerosol Harvard concentrator which produces up to a 30-fold increase in particle number and mass. Air temperature and humidity were controlled inside the chamber. The concentration of particles delivered to the chamber varied with the level of naturally occurring ambient particles in Chapel Hill air. However, a particle dilution system was used to limit the maximal particle concentration and prevent it from exceeding 600 µg/m^3^ for > 6 min at any time during exposure. A face mask was used to maximize the PM concentration inhaled by the participants. Particle mass and number concentrations at the chamber inlet were monitored continuously as described previously (Samet et al. 2009). Filter samples were also obtained and analyzed for particle mass.

*Ambulatory electrocardiography (ECG) measurements.* Ambulatory ECG data were collected for HRV analysis as described previously (Samet et al. 2009). Briefly, 12-lead Holter ECG data were collected for approximately 48 hr using a Mortara H12+ Recorder (Mortara Instrument, Milwaukee, WI). HRV indices in both the time and frequency domains and ventricular repolarization were calculated from the raw Holter ECG data using SuperECG software (version 4.0; Mortara Instrument). HRV was measured to evaluate the influence of CAP exposure on the autonomic nervous system control on the heart. Approximately 90 min before both the filtered air and CAP exposures, the participants were asked to recline quietly in a darkened room for 30 min. During the final 10 min of the resting period, data were collected and used to calculate frequency domain parameters of HRV [normalized LF (nLF), normalized HF (nHF), and the high frequency/low frequency ratio (HF/LF ratio)] and repolarization parameters. This 30-min regimen was repeated 15 min after exposure to filtered air and CAP and again the next morning to obtain Post and Follow-up measurements, respectively. Time domain HRV parameters {SDNN [standard deviation of normal to normal (NN) intervals], PNN_50_, [fraction of consecutive NN intervals that differ by more than 50 msec], RMSDD [the square root of the mean of the sum of the squares of differences between adjacent NN intervals]} were calculated from the data collected over two 24-hr periods (from 2 hr before air exposure until 0800 hours the next morning, and from 2 hr before CAP exposure until 0800 hours the next morning). The HRV parameters were determined according to established guidelines (Task Force of the European Society of Cardiology the North American Society of Pacing Electrophysiology Circulation 1996). The QT interval corrected for heart rate (QTc) was measured to determine the influence of exposure to CAP on ventricular repolarization. The interval from the onset of QRS to the peak of the T wave (QTp), the interval from the peak of the T-wave to the end of the T-wave (Tp-Te), the ratio of the transmural dispersion of repolarization relative to the total duration of repolarization (Tp-Te/QT) were measured to assess the effects of CAP exposure on spatial dispersion of repolarization.

*Blood chemistry and lipids.* Venous blood was collected 2 hr before both the filtered air and CAP exposures (“Pre”), immediately after each exposure, and again at 0800 hours the morning after CAP exposure. LabCorp (Burlington, NC) performed complete blood counts, including a differential count and a lipid panel.

*Statistical analysis.* To assess changes in biological end points between the two exposures and among the FO and OO groups, we used a two-factor (supplement and PM concentration) mixed effects model with a subject-specific random intercept. Changes in HRV, cardiac repolarization, and blood parameters were assessed at two time points: immediately and approximately 20 hr after exposure to CAP and filtered air, denoted “Post” and “Follow-up” respectively. To control for day-to-day variability, time domain measures of HRV, cardiac repolarization, blood counts, and lipids were normalized by dividing the Post and Follow-up values by the values measured before filtered air exposure (Post/Pre, Follow-up/Pre). Time domain HRV parameters were calculated once, over a 24-hr period. Changes are expressed as percent change after CAP exposure (per 100-µg/m^3^ increase in CAP) relative to change after air exposure. R statistical software (version 2.11.1; R Developement Core Team; http://www.r-project.org/) was used for analysis, and a *p*-value of < 0.05 was considered significant.

## Results

Participants’ characteristics are presented in Table 1. There were no significant differences in age, body mass index (BMI), smoking history, medication usage, BP, and serum glucose or lipids of participants assigned to the FO and OO groups. Only a small percentage of each group was taking statins or ACE (angiotensin converting enzyme) inhibitors, and no participants were taking β-adrenergic receptor blockers. Before supplementation, all participants reported low dietary intakes of foods rich in n-3 FA (data not shown). The food records were collected twice—on the second and fourth week during the supplementation period—and showed that participants assigned to FO or OO consumed similar amounts of dietary n-3 FA during the supplementation period [see Supplemental Material, Table 1 (http://dx.doi.org/10.1289/ehp.1104472)].

**Table 1:** Characteristics of the study participants before supplementation.

Characteristic	FO (n = 16)	OO (n = 13)	p-Value
Sex (male/female)		4/12		4/9		
Age (years)		57.4 ± 1.4		59.3 ± 1.1		0.32
Race (white/black)		10/6		11/2		
BMI (m2/kg)		27.6 ± 1.1		26.3 ± 1.3		0.44
Systolic BP (mmHg)		122 ± 3		123 ± 3		0.92
Diastolic BP (mmHg)		77 ± 2		77 ± 2		0.95
Heart rate (bpm)		74 ± 2		71 ± 3		0.47
Cholesterol (mg/dL)						
Total		201 ± 12		214 ± 7		0.41
LDL		117 ± 10		125 ± 9		0.57
VLDL		19 ± 2		19 ± 3		0.87
HDL		64 ± 4		70 ± 6		0.46
Triglycerides (mg/dL)		97 ± 10		94 ± 14		0.88
Glucose (mg/dL)		88 ± 3		93 ± 2		0.17
WBC (× 103/µL)		5.53 ± 0.25		5.46 ± 0.39		0.88
RBC (× 106/µL)		4.45 ± 0.12		4.76 ± 0.17		0.14
Platelets (× 103/µL)		245.9 ± 13.6		223.3 ± 12.2		0.25
Neutrophils (%)		55.9 ± 2.9		55.3 ± 2.7		0.88
Lymphocytes (%)		34.9 ± 2.9		34.6 ± 2.5		0.94
Smoking history (no. of participants)						
Nonsmokers		11		11		
Ex-smokers		5		2		
Current smokers		0		0		
Medications (no. of participants)						
Statin		1		2		
β-adrenergic receptor blockers		0		0		
ACE inhibitors		2		3		
Antidepressants		2		1		
NSAIDs		0		0		
Abbreviations: ACE, angiotensin converting enzyme; BMI, body mass index; HDL, high-density lipoprotein; LDL, low-density lipoprotein; NSAID, nonsteroidal antiinflammatory drug; RBC, red blood cells; VLDL, very low-density lipoprotein; WBC, white blood cells. Data are presented as n or mean ± SE. Two-sample t-test was used to compare the difference between the FO and OO groups.						

*Plasma FA levels.* After the 4-week supplementation period, we compared the ratio of several plasma fatty acids between the FO and OO groups. These data are shown in Table 2. Eicosapentaenoic acid levels were 6.5 times higher, linolenic acid (ALA) levels 1.4 times higher, and docosapentaenoic acid (DPA) levels 2.2 times higher in the FO group compared with the OO group. Plasma DHA levels were not different between the two groups and levels of arachidonic acid (AA) were significantly lower in the FO group than in the OO group. There was also a trend for elevation in plasma levels of oleic acid, the principal fatty acid in OO, in the OO group compared to the FO group (*p* = 0.06).

**Table 2:** Percent of total plasma fatty acids after 4 weeks of supplementation with fish oil or olive oil.

Fatty acid	FO (n = 16)	OO (n = 13)	p-Value
C16:0 palmitic acid		29.78 ± 0.52		30.19 ± 0.57		0.59
C18:1 oleic acid		15.63 ± 0.66		17.44 ± 0.61		0.06
C18:2 linoleic acid (LA)		20.82 ± 0.91		20.71 ± 0.55		0.92
C18:3 linolenic acid (ALA)		0.56 ± 0.05		0.41 ± 0.02		0.03
C20:4 arachidonic acid (AA)		7.40 ± 0.46		9.07 ± 0.44		0.02
C20:5 eicosapentaenoic acid		3.38 ± 0.33		0.52 ± 0.04		< 0.001
C22:5 docosapentaenoic acid (DPA)		1.07 ± 0.09		0.48 ± 0.04		< 0.001
C22:6 docosahexaenoic acid (DHA)		1.88 ± 0.16		1.65 ± 0.14		0.51
Data are presented as mean ± SE. Two-sample t-test was used to compare the difference between the FO and OO groups. Nonabundant fatty acids that did not differ between the two groups are not shown.						

*CAP exposure.* Particle concentrations inside the chamber varied with each exposure, and were dependant on the outside ambient air levels [see Supplemental Material, Table 2 (http://dx.doi.org/10.1289/ehp.1104472)]. Ambient PM_2.5_ concentrations on CAP exposure days ranged from 4.5 to 21.3 µg/m^3^, with an average concentration of 11.6 ± 1.0 µg/m^3^. CAP concentrations in the chamber ranged from 83 to 470 µg/m^3^ with a mean of 278 ± 19 µg/m^3^. Particle number concentrations in the chamber ranged from 22,634 to 1,970,748 particles/cm^3^ with a mean of 302,515 ± 77,603 particles/cm^3^. Particle mass concentrations in the filtered air in the chamber varied from 0 to 0.40 µg/m^3^, with a mean of 0.21 µg/m^3^. There was no significant difference in mean CAP concentrations between the two groups (301 ± 25 µg/m^3^ in the FO group; 269 ± 37 µg/m^3^ in the OO group). Similarly, mean particle number concentrations were not significantly different (305,800 ± 120,900 particles/cm^3^ in the FO group; 322,500 ± 97,510 particles/cm^3^ in the OO group).

**Figure 2:**
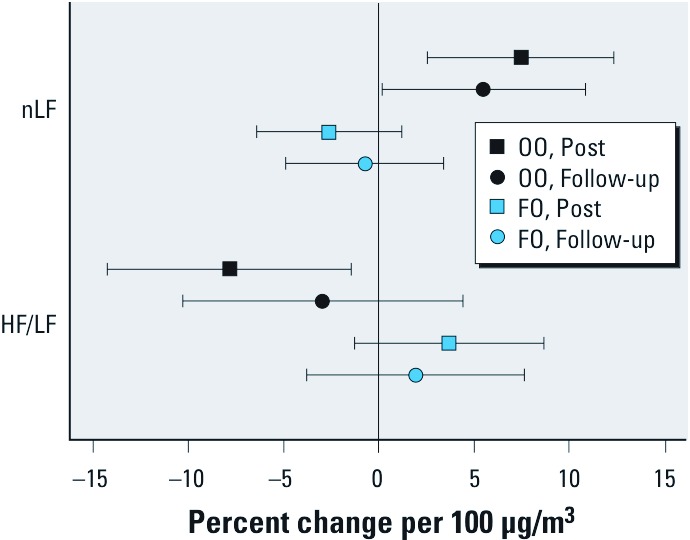
Effect of CAP exposure on frequency domain indices of HRV. nLF HRV and HF/LF ratio were analyzed in the ECG recordings of participants at rest before, immediately after exposure to filtered air and CAP (Post), and again the next morning (Follow-up) as described in “Methods.“ Data shown are average changes per 100-µg/m^3^ increase in CAP relative to the filtered air and 95% confidence intervals.

*HRV and cardiac repolarization.* Cardiac repolarization and HRV frequency domain parameters measured 2 hr before filtered air exposure (Pre) were not significantly different between the FO and OO groups (data not shown). nLF HRV was significantly increased in the OO group immediately after CAP exposure, and the change persisted for at least 20 hr (Figure 2 and Table 3). The HF/LF ratio was significantly decreased in participants supplemented with OO immediately after CAP exposure, but the decrease was no longer significant by the next morning (Figure 2). In contrast, participants taking FO did not show significant CAP-induced changes in nLF or HF/LF ratio at either time point (Figure 2). Time domain parameters and HF HRV did not change after CAP exposure in participants supplemented with either type of oil (data not shown). [See Supplemental Material, Table 3 (http://dx.doi.org/10.1289/ehp.1104472) for individual values before and after each exposure for the Holter parameters that changed.]

**Table 3:** Percent change from the filtered air exposure for individual end points per 100 µg/m3 of CAP.

FO (n = 16)			OO (n = 13)		
End points	Post-CAP	Follow-up	Post-CAP	Follow-up
HRV								
nLF		–2.62 ± 1.85		–0.74 ± 2.02		7.41 ± 2.38*		5.47 ± 2.60*
HF/LF		3.67 ± 2.42		1.89 ± 2.77		–7.87 ± 3.12*		–2.97 ± 3.58
QT								
QTc		0.05 ± 0.11		0.08 ± 0.20		0.12 ± 0.15		0.58 ± 0.26*
QTp		0.22 ± 0.33		0.23 ± 0.43		0.44 ± 0.42		1.70 ± 0.55*
Tp-Te		0.65 ± 0.34		1.17 ± 0.48*		–0.48 ± 0.44		1.13 ± 0.62
Tp-Te/QT		0.32 ± 0.43		0.72 ± 0.46		–0.66 ± 0.56		–0.44 ± 0.59
Lipids								
VLDL		1.54 ± 1.95		–5.22 ± 2.97		7.68 ± 2.55*		6.33 ± 3.82
LDL		–0.30 ± 0.81		1.08 ± 0.89		0.54 ± 1.05		–1.40 ± 1.15
HDL		–0.19 ± 0.72		0.59 ± 0.68		–0.46 ± 0.93		–1.10 ± 0.87
Triglycerides		1.27 ± 1.93		–5.32 ± 2.96		7.40 ± 2.52*		6.00 ± 3.81
Total cholesterol		0.13 ± 0.50		0.45 ± 0.61		1.10 ± 0.65		–0.57 ± 0.79
Blood cells								
WBC		0.01 ± 1.04		2.81 ± 1.42		0.06 ± 1.36		–2.06 ± 1.83
RBC		–0.35 ± 0.38		0.21 ± 0.43		–0.12 ± 0.49		–0.75 ± 0.55
Platelets		0.26 ± 0.48		0.59 ± 0.69		–0.41 ± 0.62		–0.30 ± 0.88
Neutrophils		–0.47 ± 0.91		–0.02 ± 1.17		–1.10 ± 1.18		–3.05 ± 1.50*
Lymphocytes		0.35 ± 1.42		–0.83 ± 2.39		1.27 ± 1.85		4.47 ± 3.08
Monocytes		–0.94 ± 3.12		1.02 ± 1.99		0.60 ± 4.02		3.13 ± 2.56
Parameter estimates are summarized as mean ± SE. *p < 0.05, compared to filtered air.								

**Figure 3:**
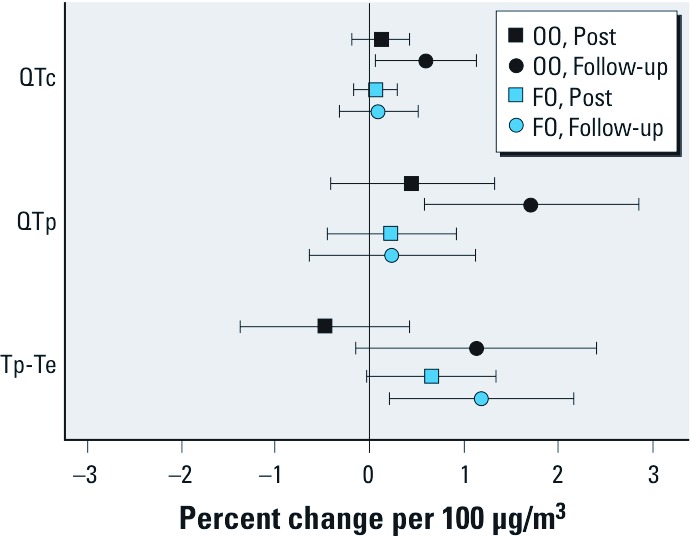
Effect of CAP exposure on indices of cardiac repolari­zation. QTc, QTp, and Tp-Te were analyzed in ECG recordings of participants at rest before, immediately after exposure to filtered air and CAP (Post), and again the next morning (Follow-up) as described in “Methods.“ Data shown are average changes per 100-µg/m^3^ increase in CAP relative to the filtered air and 95% confidence intervals.

The duration of the QT interval (QTc and QTp) was prolonged 20 hr after CAP exposure in only the OO group (Figure 3 and Table 3). Conversely, the transmural dispersion of repolarization measure Tp-Te was increased by CAP exposure in only the FO group 20 hr after exposure, although there was a trend (*p* = 0.07) for an increase in the OO group (Figure 3). The ratio of Tp-Te/QT was not altered by CAP exposure in either the FO or OO groups.

**Figure 4:**
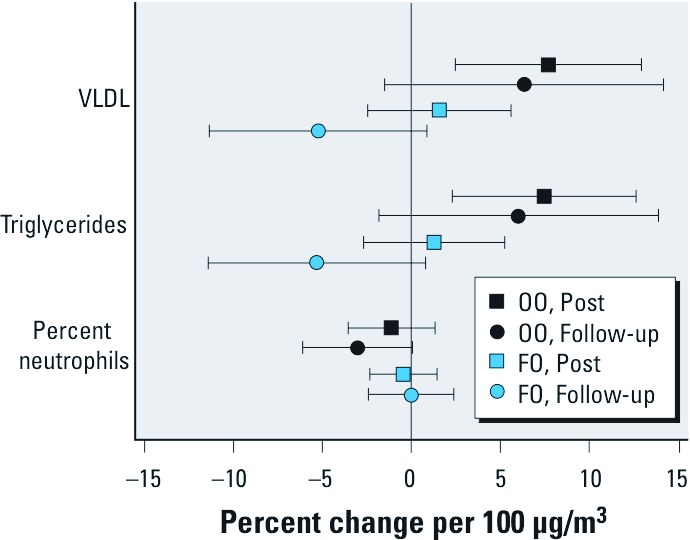
Effect of CAP exposure on plasma lipid levels and blood percent of neutrophils. Blood percent of neutrophils, plasma triglycerides and VLDL were measured before, immediately after exposure to filtered air and CAP (Post), and again the next morning (Follow-up) as described in “Methods.“ Data shown are average changes per 100-µg/m^3^ increase in CAP relative to the filtered air and 95% confidence intervals.

*Blood cells and lipids.* Inhalation of CAP did not cause significant changes in most blood cell types in either supplement group, as shown in Table 3. However, the percentage of neutrophils was significantly decreased 20 hr after CAP exposure in the OO group, but not in the FO group (Figure 4).

Blood lipid levels in the participants changed after 4 weeks of dietary supplementation: Very low-density lipoprotein (VLDL) and triglyceride levels were higher in both the FO and OO groups after supplementation, high-density lipoprotein (HDL) levels were lower in both groups, and low-density lipoprotein (LDL) levels were lower in the OO group [see Supplemental Material, Table 4 (http://dx.doi.org/10.1289/ehp.1104472)]. Despite these changes that occurred in each group during the 4-week supplementation period, there were no statistical difference in blood lipid levels between the FO and OO groups at the end of the 4-week supplementation period except for VLDL in the OO group, where levels were significantly increased. Further data analysis showed that there was no significant difference in these markers after filtered air exposure compared to pre-air levels in either supplementation group (see Supplemental Material, Table 3).

However, exposure to CAP caused an immediate increase in triglyceride and VLDL in the OO group, but not in the FO group (Figure 4 and Table 3); these changes were no longer significant by the next morning. There was a nonsignificant trend for an increase in total cholesterol immediately after CAP exposure in the OO group (p = 0.09) but not the FO group.

## Discussion

In this study, a brief exposure to inhaled CAP resulted in significant autonomic, cardiac electrophysiological, and lipid changes in healthy middle-aged participants taking OO supplements but not in those supplemented with FO, suggesting that n-3 FA is protective against the physiological and biochemical effects of PM that are thought to underlie adverse health outcomes described in air pollution epidemiological studies.

Because of the number of persons worldwide who reside in areas with high air pollution, a number of previous studies have sought to identify commonly available agents that could blunt the adverse effects associated with air pollution exposure. Antioxidants and anti-inflammatory agents have been evaluated as strategies to block the respiratory effects of ambient air pollution exposure [see review by Romieu et al. (2008)]. We have previously shown that vitamin C and E supplementation was effective in blunting the decrements in lung functions but not the inflammatory responses to ozone in healthy adults (Samet et al. 2001). Antioxidant statins were reported to blunt the HRV effects of air pollution exposure in healthy aged adults who were genetically susceptible to oxidative stress [*GSTM1* (glutathione *S*-transferase mu 1) null) (Schwartz et al. 2005).

A number of studies have shown that n-3 FA can reduce mortality associated with cardiovascular disease and that supplementation with n-3 FA might protect vulnerable populations from the adverse health effects associated with air pollution exposure. The American Heart Association (AHA) scientific statement states that supplementation with n-3 FA decreases cardiac and all-cause mortality in persons with and without coronary heart diseases and that higher doses of n-3 FA can lower serum triglyceride levels (Kris-Etherton et al. 2002). Romieu et al. (2005) reported that 2 g/day of FO supplementation appeared to blunt the HRV changes associated with air pollution exposure in nursing home residents, resulting in significantly diminished time and frequency domain HRV changes associated with daily indoor PM_2.5_ exposure in participants, but not in participants supplemented with soy oil. Our findings for changes measured after controlled CAP exposures were consistent with those reported by Romieu et al. (2005) In addition, our study examined effects on a wider range of cardiovascular end points, including cardiac repolarization indices and blood lipid levels.

HRV is a marker of autonomic nervous system control on the heart. Decreased HRV has been associated with increased mortality from sudden death and ventricular arrhythmia in both healthy and diseased individuals (Kleiger et al. 1987; Lahiri et al. 2008; Tsuji et al. 1996). Air pollution exposure is associated with decreases in HRV in older adults in both epidemiological and controlled human exposure studies (Devlin et al. 2003; Gold et al. 2000; Pope et al. 2004). LF power has been used as a marker of sympathetic modulation of heart rate, while the ratio of LF/HF power has been used as an index of “sympatho-vagal balance” (Pagani et al. 1986). Our data show that a brief exposure to CAP in middle-aged participants supplemented with OO altered the cardiac autonomic activity by increasing nLF and decreasing the ratio of HF/LF of HRV, and that the changes in nLF persisted for at least 20 hr. These findings are consistent with a previous study demonstrating that CAP exposure alters the sympatho-vagal balance (Devlin et al. 2003; Samet et al. 2009) by increasing the sympathetic input to the cardiovascular system, which might result in cardiovascular stress including changes in heart rate, cardiac contractility, high levels of circulating catecholamines, and vasoconstriction.

Epidemiological (Mozaffarian et al. 2008) and clinical (O’Keefe et al. 2006) studies have shown a positive association between cellular content of n-3 FA and more favorable HRV measures. In the present study, plasma eicosapentaenoic acid and DPA concentrations were significantly higher in the FO group than in the OO group after 4 weeks of supplementation, suggesting that one or both of these FAs contributes to the protective effect observed in this study. The mechanism by which n-3 FA influences HRV is thought to involve the modulation of adrenergic-mediated baroreceptor activity or enhancement of vagal activity (Mozaffarian et al. 2008; O’Keefe et al. 2006). Our findings suggest that n-3 FA improves autonomic balance by suppressing PM-induced increases in sympathetic nervous activity.

QT and QTc intervals have been used to assess ventricular repolarization. Prolongation of repolarization may indicate the likelihood of increased arrhythmia vulnerability. Studies have shown that air pollution exposure can prolong cardiac repolarization. Epidemiological investigation of patients with coronary artery disease (Hampel et al. 2010) and controlled exposure studies of healthy volunteers (Samet et al. 2009) have shown increased QT intervals after exposure to PM, suggesting that PM exposure may increase the risk of atrial and ventricular arrhythmias. Furthermore, prolonged Tp-Te interval and Tp-Te/QT ratio have been proposed as markers of ventricular arrhythmogenesis (Watanabe et al. 2004; Yamaguchi et al. 2003) and a recent controlled exposure study showed that exposure to CAP and ozone increased Tp-Te in healthy adults (Sivagangabalan et al. 2011). Clinical studies have suggested that fish or n-3 FA intake decreases the likelihood of a long QT interval (Mozaffarian et al. 2006) and decreases susceptibility of the heart to atrial and ventricular arrhythmia (Albert et al. 2002). *In vitro* studies have reported that n-3 FA directly affect cardiac electrophysiology by reducing myocyte excitability and cytosolic calcium fluctuations via inhibition of the voltage-dependent sodium and L-type calcium channels and sodium/calcium exchange currents (Xiao et al. 1995, 1997). In the present study, supplementation of n-3 FA blocked the PM-induced QT prolongation but not the dispersion of ventricular repolarization (Tp-Te).

There were significant increases in triglycerides and VLDL immediately after CAP exposure in participants supplemented with OO, a finding that is consistent with our previous report showing that exposure to PM elevated triglycerides and VLDL in asthmatic adults (Yeatts et al. 2007). In fact, PM exposure has been associated with elevated levels of lipid peroxidation products in human blood cells, plasma, and urine (Moller and Loft 2010). Ambient air pollution exposure has been associated with the development of atherosclerosis in elderly men (Künzli et al. 2005) and apoE^-/-^ mice (Sun et al. 2005). Therefore, our data suggest the possibility that the increased triglyceride and VLDL levels associated with PM exposure is a potential mechanism for atherosclerosis. Given that the hypotriglyceridemic effects of n-3 FA are well established, and the U.S. Food and Drug Administration (FDA) has approved an n-3 FA ethyl ester for the treatment of very high triglyceride levels (Bays 2006), it is not surprising that n-3 FA appeared to attenuate CAP-induced increases in triglycerides and VLDL immediately after exposure in this study. The mechanism for these lipid-lowering effects seems to involve the activation of peroxisome proliferator-activated receptors (Berger and Moller 2002).

The AHA recommends that everyone eat at least two meals of oily fish per week, and that those with a family or personal history of coronary heart disease supplement their diet with FO tablets containing 600 mg eicosapentaenoic acid and DHA daily to prevent cardiovascular disease. The dose of FO supplementation in the present study was consistent with the FDA recommendation of 3.5 g FO/day. Supplementation with 3 g FO/day (about 2,000 mg eicosapentaenoic acid plus DHA) appeared to have a beneficial, attenuating effect on the changes in HRV and lipid levels induced by inhalation of PM_2.5_ and PM_0.1_ air pollution particles in healthy middle-aged adults.

There are limitations to this study. This study did not use a standard crossover design in which exposure to air and CAP was randomized. A nonrandom design was used because plasma concentrations of fatty acids used in the supplements did not reach equilibrium even after 4 weeks. To ensure that each participant’s FA levels were comparable during their air and CAP exposures, both exposures were done in close proximity with one another (a day apart). However, inhaled particles are not cleared from the lung for days, and thus CAP studies separate air and CAP exposures by 2–4 weeks to account for the long washout period of particles. Because of these concerns, we chose to always expose participants to air first. In addition, conclusions derived from the small number of participants included in the study may not be applicable to the population as a whole.

## Conclusions

A short exposure to CAP induced acute changes in HRV, blood neutrophils, and lipids that were attenuated in healthy middle-aged adults supplemented with FO, but not in those supplemented with OO. Our findings suggest that n-3 FA supplements may offer protection against the adverse cardiac and lipid effects associated with air pollution exposure.

## Supplemental Material

(205 KB) PDF
