# Supplementary material for: Omega-3 Fatty Acid Supplementation Appears to Attenuate Particulate Air Pollution–Induced Cardiac Effects and Lipid Changes in Healthy Middle-Aged Adults
Source: Environ Health Perspect. 2012 Apr 19;120(7):952–7. doi: 10.1289/ehp.1104472 (PMC3404661; doi:10.1289/ehp.1104472)
Supplement: (205 KB) PDF [file ehp.1104472.s001.pdf]

Supplemental Material

Omega-3 Fatty Acid Supplementation Appears to Attenuate Particulate Air Pollution Induced Cardiac Effects and Lipid Changes in Healthy Middle-Aged Adults

Haiyan Tong[1], Ana G. Rappold[1], David Diaz-Sanchez[1], Susan E. Steck[2], Jon Berntsen[3], Wayne E. Cascio[1], Robert B. Devlin[1], and James M. Samet[1]

[1]Environmental Public Health Division, NHEERL, US Environmental Protection Agency, Research Triangle Park, NC 27711; [2]Department of Epidemiology and Biostatistics, University of South Carolina, Columbia, SC 29208; [3]TRC Environmental Corporation, Raleigh, NC 27606.

Corresponding author: Haiyan Tong, MD, PhD.
Environmental Public Health Division
NHEERL, US Environmental Protection Agency
Mail Code: 58D
109 TW. Alexander Dr.
Research Triangle Park, NC 27709
Phone: (919) 966-4993
Fax: (919) 966-6271
E-mail: tong.haiyan@epa.gov

Table of contents

Supplemental Table 1. Estimated dietary fatty acids intake.
Supplemental Table 2. CAP exposure data of each subject.
Supplemental Table 3. Individual variables before and after exposure.
Supplemental Table 4. Plasma lipids before and after supplementations.

Supplemental Material, Table 1. Estimated dietary fatty acids intake.

Fatty acids	Fish oil (n=16)	Olive oil (n=13)	P value
Oleic acid (g/d)	19.59±1.86	20.88±1.85	0.63
Linoleic acid (g/d)	10.71±1.22	11.34±1.23	0.72
Linolenic acid (g/d)	1.11±0.15	1.26±0.13	0.46
Arachidonic acid (g/d)	0.13±0.02	0.10±0.01	0.28
Eicosapentaenoic acid (g/d)	0.083±0.045	0.013±0.004	0.18
Docosapentaenoic acid (g/d)	0.025±0.007	0.011±0.002	0.13
Docosahexaenoic acid (g/d)	0.082±0.030	0.042±0.011	0.26
Total omega-3 fatty acid (g/d)	1.30±0.15	1.33±0.12	0.88

The data are the average of all 6 days of intake from each subject during the supplementation period. Dietary intake estimates were derived by having a registered dietitian enter the diet records into NDSR (Nutrition Data System for Research) which calculates nutrient values using a food composition database containing over 18,000 food items. Data are means ± SE. Student's t-test was used to compare the difference between the fish oil and olive oil groups.

Supplemental Material, Table 2. CAP exposure data of each subject[a,b]

Subject	Supplementation	Date	Fine Particle Mass Concentration (μg/m^3)	TEOM® Mass Concentration (μg/m^3)	Particle Concentration (#/cc)
1	FO	6/30/2009	83.05	88.42	43,359
2	FO	7/7/2009	230.02	234.91	65,528
3	OO	7/14/2009	299.51	272.31	460,621
4	OO	7/21/2009	298.76	300.36	61,291
5	FO	7/28/2009	317.68	312.23	145,762
6	OO	8/4/2009	205.76	206.58	42,240
7	OO	8/11/2009	192.78	194.80	60,702
8	FO	8/18/2009	n/a	284.90	44,372
9	OO	8/25/2009	429.27	451.22	93,879
10	FO	12/15/2009	220.08	201.63	66,992
11	FO	9/15/2009	354.37	331.09	71,199
12	OO	9/29/2009	198.46	186.43	1,181,572
13	FO	10/20/2009	318.83	276.81	692,176
14	OO	10/27/2009	153.52	126.07	79,889
15	FO	11/3/2009	344.72	324.33	404,506
16	OO	11/24/2009	79.25	75.70	151,000
17	OO	1/5/2010	206.18	192.99	655,807
18	FO	2/9/2010	444.64	358.41	550,049
19	FO	5/11/2010	249.47	239.67	164,523
20	OO	3/23/2010	147.09	127.44	630,426
21	FO	4/13/2010	430.03	361.11	1,970,751
22	OO	4/20/2010	335.76	275.43	572,941
23	FO	4/27/2010	257.12	221.94	206,689
24	FO	5/4/2010	326.26	278.35	173,540
25	OO	6/8/2010	258.88	256.93	142,609
26	FO	6/15/2010	470.30	461.58	150,640
27	FO	6/29/2010	322.24	534.05	120,316
28	FO	7/13/2010	177.49	174.67	22,634
29	OO	8/10/2010	413.13	394.25	59,396

[a]Data obtained from PCS exposure records and human studies database EES performance summary report; [b]Measured at the inlet to the chamber. FO: fish oil supplementation; OO: olive oil supplementation; n/a: no filter data for this run.

Supplemental Material, Table 3. Individual variables before and after exposure.

Variables	Fish oil (n=16)					Olive oil (n=13)				
	Filtered Air		CAP			Filtered Air		CAP		
	Pre	Post	Pre	Post	FU	Pre	Post	Pre	Post	FU
nHF	48.2±1.9	47.8±1.9	45.5±2.1	45.7±2.1	43.7±2.7	49.4±1.6	50.9±2.0	46.0±1.5	45.4±1.5	46.0±1.9
nLF	30.7±1.8	33.1±1.7	31.4±1.7	33.0±1.8	31.8±1.7	35.0±1.6	33.1±1.4	34.6±1.9	36.5±1.6	36.3±1.2
HF/LF	1.66±0.13	1.51±0.10	1.52±0.10	1.47±0.11	1.45±0.12	1.46±0.11	1.58±0.10	1.39±0.10	1.28±0.08	1.30±0.09
QTc (ms)	425.3±4.3	424.5±4.6	423.7±3.8	424.8±3.7	423.9±3.2	416.2±3.5	416.7±3.6	413.4±3.8	416.1±3.7	416.6±3.9
QTp (ms)	318.2±5.0	325.1±5.4	312.4±4.9	320.4±5.7	309.9±5.9	321.5±3.1	321.8±3.7	311.2±3.1	315.2±4.1	314.8±5.7
Tp-Te (ms)	96.0±2.1	94.0±2.4	93.6±1.9	93.7±2.4	95.8±2.9	97.7±2.2	98.8±2.5	96.0±2.2	96.1±1.8	97.8±2.5
Tp-Te/QT	0.232±0.004	0.224±0.005	0.230±0.004	0.227±0.005	0.236±0.007	0.233±0.004	0.235±0.005	0.236±0.005	0.233±0.005	0.238±0.006
HR_{mean} (bpm)	64.0±2.0	61.1±2.1	66.2±2.4	63.7±2.3	67.6±2.7	58.7±1.6	57.8±1.6	62.1±1.4	60.8±1.7	60.7±1.8
T-cholesterol (mg/dl)	196±12	204±12	192±11	199±11	186±10	205±7	205±8	201±8	208±8	195±7
LDL (mg/dl)	113±10	120±9	110±9	114±9	108±9	114±7	112±7	113±7	113±7	106±6
VLDL (mg/dl)	29±5	30±5	29±4	30±4	28±4	44±7	45±7	42±7	44±6	43±7
HDL (mg/dl)	59±4	61±4	57±4	58±4	54±4	65±5	65±5	64±5	64±5	61±5
Triglyceride (mg/dl)	121±17	118±15	127±15	129±15	120±13	131±11	141±15	123±11	154±18	142±19
WBC ($x10^3/\mu l$)	5.5±0.3	6.3±0.3	5.2±0.2	6.0±0.1	5.4±0.2	5.2±0.3	6.2±0.3	5.6±0.5	6.4±0.3	5.4±0.3
Neutrophils (%)	53±2	55±2	53±2	54±2	54±2	56±3	58±3	60±3	58±3	57±3
Lymphocytes (%)	37±2	36±2	37±2	37±2	36±2	33±2	31±2	30±3	31±3	33±3
Monocytes (%)	7.1±0.5	7.5±0.6	6.9±0.5	7.1±0.4	7.3±0.5	7.2±0.4	7.4±0.5	6.6±0.4	7.3±0.5	6.8±0.5
RBC ($x10^6/\mu l$)	4.5±0.1	4.5±0.1	4.4±0.1	4.4±0.1	4.3±0.1	4.5±0.1	4.6±0.1	4.5±0.1	4.6±0.1	4.4±0.1
Platelets ($x10^3/\mu l$)	253±14	260±14	250±14	260±15	248±14	232±12	243±13	233±11	243±12	231±12

Values are means ± SE. Pre: pre-exposure; Post: post-exposure; FU: follow up; nHF: normalized high frequency; nLF: normalized low frequency; HF/LF: high frequency/low frequency ratio; QTc: heart rate corrected QT interval; QTp: peak of QT interval ; Tp-Te: the interval from the peak of the T-wave to the end of the T-wave; Tp-Te/QT: ratio of Tp-Te over the total duration of QT; HR_{mean}: mean heart rate; T-cholesterol : total cholesterol; VLDL: very low- density lipoprotein; LDL: low density lipoprotein; HDL: high density lipoprotein; WBC: white blood cells; RBC: red blood cells.

Supplemental Material, Table 4. Plasma lipids levels before and after supplementations.

	Fish oil (n=16)			Olive oil (n=13)		
Lipids	Before	After	p value	Before	After	p value
Total cholesterol (mg/dl)	201±12	196±12	0.27	214 ± 7	205±7	0.08
LDL (mg/dl)	117 ± 10	113±10	0.35	125 ± 9	114±7	0.04
VLDL (mg/dl)	19 ± 2	29±5	0.02	19 ± 3	44±7	0.01
HDL (mg/dl)	64 ± 4	59±4	0.02	70 ± 6	65±5	0.04
Triglyceride (mg/dl)	97 ± 10	121±17	0.05	94 ± 14	131±11	0.001

Values are means ± SE. Before: before supplementation: After: after 4 weeks of supplementation; LDL: low density lipoprotein; VLDL: very low- density lipoprotein; HDL: high density lipoprotein; Student's t-test was used to compare the difference between the before and after supplementation levels.
